# The Role of ATRA, Natural Ligand of Retinoic Acid Receptors, on EMT-Related Proteins in Breast Cancer: Minireview

**DOI:** 10.3390/ijms222413345

**Published:** 2021-12-12

**Authors:** Pavel Bobal, Marketa Lastovickova, Janette Bobalova

**Affiliations:** 1 Department of Chemical Drugs, Faculty of Pharmacy, Masaryk University, 612 00 Brno, Czech Republic; bobalp@pharm.muni.cz; 2 Institute of Analytical Chemistry of the CAS, v. v. i., 602 00 Brno, Czech Republic; lastovickova@iach.cz

**Keywords:** breast cancer, EMT, protein, ATRA

## Abstract

The knowledge of the structure, function, and abundance of specific proteins related to the EMT process is essential for developing effective diagnostic approaches to cancer with the perspective of diagnosis and therapy of malignancies. The success of all-*trans* retinoic acid (ATRA) differentiation therapy in acute promyelocytic leukemia has stimulated studies in the treatment of other tumors with ATRA. This review will discuss the impact of ATRA use, emphasizing epithelial-mesenchymal transition (EMT) proteins in breast cancer, of which metastasis and recurrence are major causes of death.

## 1. Introduction

In the last two decades, the total number of people diagnosed with cancer has almost doubled, from an estimated 10 million in 2000 to 19.3 million in 2020. Today, every 5 people around the world suffer from cancer during their lifetime. Cancer deaths have also increased, from 6.2 million in 2000 to 10 million in 2020. More than one in six deaths is caused by cancer. Breast cancer has now overtaken lung cancer as the world’s most commonly diagnosed cancer, according to statistics released by the International Agency for Research on Cancer (IARC) in December 2020 [1]. Approximately 1 in 8 women (13%) will be diagnosed with invasive breast cancer during their lifetime, and 1 in 39 women (3%) will die of breast cancer [2]. That’s why there is a growing need to learn about this disease as much as possible and the need to seek new and more effective drugs. In addition, breast cancer usually does not cause any symptoms if the tumor is small and is easiest to treat. Therefore, early cancer detection is essential for an accurate diagnosis to reduce the possibility of metastasis and relapse. The use of up-to-date analytical proteomic techniques, especially current chromatographic or electrophoretic separation methods together with mass spectrometry (MS), and the development of new analytical strategies is critical for the characterization of cancer cells and especially for identifying new diagnostic and prognostic biomarkers, which have mainly protein characters. The prominent role of proteomics is to identify biomarkers for early cancer screening and predict therapeutic response [3,4,5].

Retinoids, the group of vitamin A derivatives, are currently receiving considerable attention because their properties predispose it to become an anticancer agent, as confirmed by the growing body of evidence highlighting the compound’s anticancer activity. All-*trans* retinoic acid (ATRA) is administered orally in the first-line treatment of acute promyelocytic leukemia (APL) [6] in adults and neuroblastoma (NB) in children [7]. As the promising results obtained in these diseases have not yet translated to the solid tumor clinic, there remains a large room for further in-depth studies.

### 1.1. Aim of the Study

The review focuses on ATRA properties, emphasizing epithelial-mesenchymal transition (EMT) proteins in breast cancer. The core of the review summarizes the current knowledge on the effectiveness of the ATRA system in the EMT process, in which epithelial cells lose their cell polarity and acquire migratory and invasive properties to become mesenchymal stem cells. Based on the positive results, we emphasize the necessary implementation of ATRA in research, focusing on the anticancer approach.

### 1.2. Source of the Data

Data were recovered from the biomedical literature by the use of “ATRA” and “EMT” and “breast cancer” or other associated terms as either a keyword term in searches of the Web of Science bibliographic database. In the particular part, focusing on the anticancer effects of ATRA, we mainly emphasize the most recent scientific papers from the years 2015–2021.

## 2. Molecular Subtypes of Breast Cancer

Breast cancer treatment has advanced significantly in the past five years. The principles of breast cancer therapy follow a curative purpose and must be determined in a multidisciplinary sense, taking into account molecular subtype and loco-regional tumor load. Advancements in therapeutic strategies make the prospect of long-term disease control in metastatic breast cancer an increasing reality.

**Figure 1:**
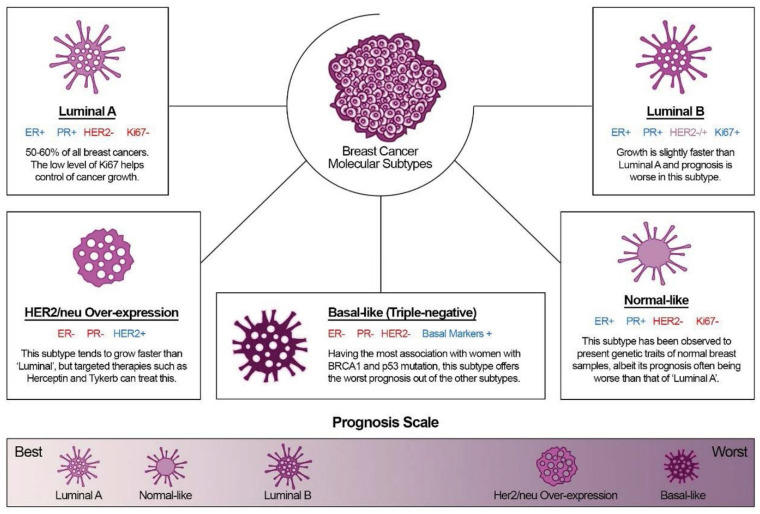
Breast cancer and its five molecular subtypes: prognosis of the disease. Reprinted with permission from [8]. Copyright 2018 ClinMed International Library.

It is well known that different combinations of the presence of estrogen receptor (ER), progesterone receptor (PR), human epidermal growth factor receptor 2 (Her2) status, Ki67 protein and tumor grade define five basic molecular subtypes of breast cancer, luminal A, luminal B Her2+/luminal B Her2−, basal/triple-negative, normal-like, and Her2-enriched [8,9]. The status within each subtype is summarized in Figure 1.

1. Luminal A (ER+/PR+/HER2−/Ki67−): This is the most common type of breast cancer and tends to be slower-growing and less aggressive than other subtypes. Luminal A tumors are associated with the most favorable prognosis in part because they are usually responsive to hormonal therapy [10]. Tumors also show good differentiation, low grade (1 or 2), and the percentage of their recurrence is low [11]. In addition, the low level of Ki67 protein helps control of cancer growth [8].
2. Luminal B (ER+/PR+/HER2− or HER2+/Ki67+): This is a relatively small subgroup of tumors that proliferate significantly more, are less differentiated and express hormone receptors. In addition, this subtype was initially characterized clinically as always being positive for HER2, but more recently has been defined as being highly positive for the protein Ki67 and/or HER2 [12]. Luminal B breast cancers have higher histological than luminal A and recur more often.
3. Basal-like (ER−/PR−/HER2−): These cancers are also called triple-negative because they lack these receptors. This subtype, which has the most significant association with women with the BRCA1 and p53 gen mutations, offers the worst prognosis of the other subtypes, in part because treatment advances have lagged behind other molecular subtypes [13]. The majority (about 75%) of triple-negative breast cancers fall into the basal-like subtype defined by gene expression profiling. Proliferative activity is significant. Patients of luminal A and basal subtype form the regional lymph node metastases less frequently [14].
4. HER2-enriched (ER−/PR−/HER2+): In the past, this subtype had the worst prognosis; however, the widespread use of targeted therapies for HER2+ cancers have substantially improved outcomes for these patients [15].
5. Normal-like (ER+/PR+/HER2−/Ki67−): This subtype has been found to exhibit the genetic characteristics of normal breast samples, although its prognosis is often worse than the luminal A prognosis [8].

However, some data suggest that the current classification scheme for breast tumors may not fully capture cancer’s genetic and molecular status. The revised classification will allow the more accurate treatment of cancer. Today, the search for better classifiers of tumors is significantly focused on applying omic approaches, which can analyze thousands of gene sequences, gene transcripts, or proteins in a single experiment. Bouchal and colleagues [9] recently demonstrated and confirmed the suitability of sequential windowed acquisition of all theoretical fragment ion mass spectra approach (SWATH-MS) for proteotyping of human tumor samples and also identified key proteins for the classification of breast tumors. Proteins that contribute most strongly to proteotype-based classification include inositol polyphosphate-4-phosphatase, type II (INPP4B), cyclin-dependent kinase 1 (CDK1), and receptor tyrosine kinase 2 (ERBB2) are associated with estrogen receptor (ER) status, HER2 status tumor, and grade status. Although more data are needed to validate classifiers, the results suggest that proteotype-based classification may improve the current conventional classification of breast tumors and thus provide adequate treatment.

## 3. Epithelial-Mesenchymal Transition

**Figure 2:**
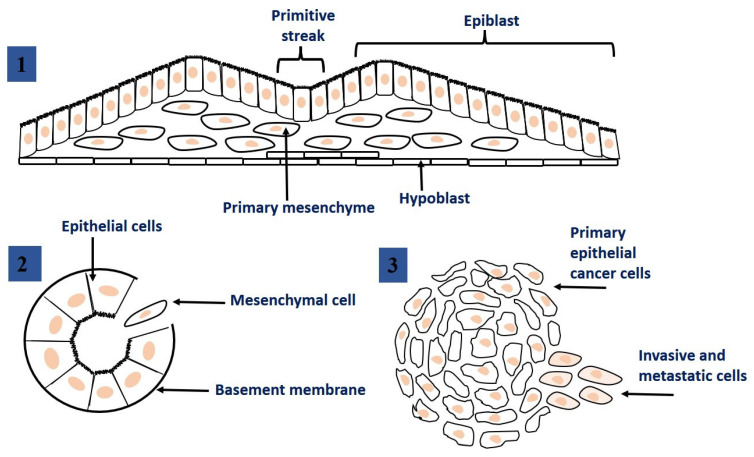
Epithelial to mesenchymal transition: the process of transformation of epithelial cells into mesenchymal cells. 1. EMT related to implantation, embryo formation, and organ development. 2. EMT related to cancer progression and metastasis. 3. EMT associated with tissue regeneration and organ fibrosis. This figure was adapted from ref. [17].

The epithelial-mesenchymal transition (EMT) is a dynamic process during which epithelial cells lose their cellular polarity and phenotypic properties and acquire mesenchymal cell properties. EMT process also allows cells to disrupt the basement membrane and invade neighboring tissues or distant organs [16]. EMT occurs naturally in the body, for example, during tissue regeneration or embryogenesis. Nevertheless, EMT process has been suggested that may be closely linked to the acquisition of aggressive properties by tumor cells, facilitating the initial stages of metastasis. EMTs occur in three different biological subtypes, which have very different functional consequences [17]. Figure 2 shows different types of EMT, and at the same time, Table 1 provides an overview of some of the most common markers that demonstrate these subtypes.

1. EMTs associated with implantation, embryo formation, and organ development are organized to generate different cell types that share common mesenchymal phenotypes. These type 1 EMTs can generate mesenchymal cells that have the potential to subsequently undergo a reverse process—a mesenchymal-epithelial transition (MET) to generate secondary epithelium.
2. Type 2 EMTs are associated with tissue regeneration and organ fibrosis. Organic fibrosis, which occurs in many epithelial tissues, is mediated by inflammatory cells and fibroblasts that release various inflammatory signals. Reliable markers for the characterization of mesenchymal products generated by EMT, which occur during the development of fibrosis in various organs, are the following proteins: fibroblast-specific protein 1, a class S100 of the cytoskeletal protein, α-SMA, and collagen I [41,42].
3. Type 3 EMTs are associated with cancer progression and metastasis. In the case of this EMT, the cancer cells on the invasive anterior side of the tumors transform into a mesenchymal phenotype. Many in vivo as well as in vitro experiments have shown that cancer cells can acquire a mesenchymal phenotype and express mesenchymal protein markers such as smooth muscle alpha-actin (α-SMA), fibroblast specific protein 1 (FSP1), vimentin, and desmin [43].

The incomplete EMT status in cancer cells allows them to possess more transient states and to express mixed epithelial and mesenchymal genes, so these cells can be more aggressive compared to cells with the complete EMT phenotype [44]. Cancer cells affecting metastases are similar to the epithelium and can be identified as morphologically and molecularly derived from the primary tumor. For this reason, cancer cells must reverse the mesenchymal phenotype of reverse EMT, a process known as the mesenchymal-epithelial junction (MET) [45].

**Figure 3:**
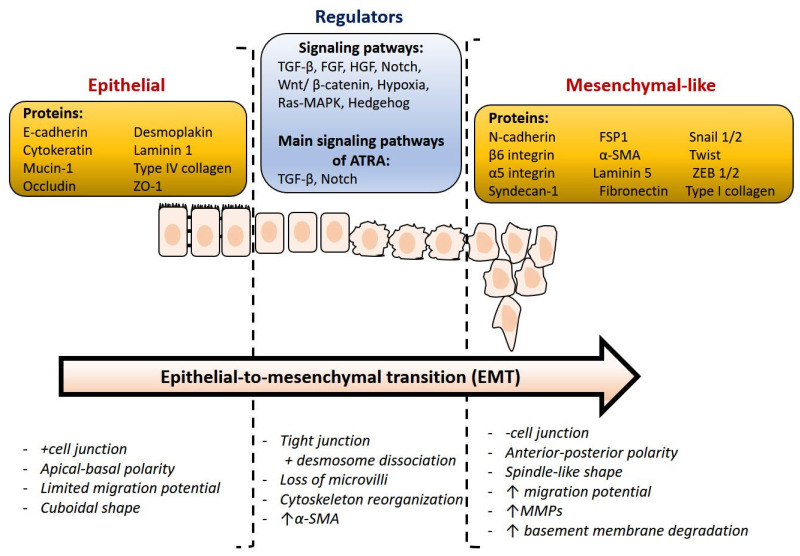
Overview of relevant markers and main molecular changes during epithelial-to-mesenchymal transition. This figure was adapted from ref. [27].

At present, it is relevant to identify some essential proteins that address important, still unanswered questions. The downregulation of epithelial markers and the upregulation of mesenchymal protein markers are both characteristics of EMT (Figure 3). A critical molecular feature of this process is the downregulation of the E-cadherin expression. E-cadherin is a key protein in cell polarity and epithelial organization. The reduction or loss of E-cadherin has become one of the hallmarks of EMT and was frequently associated with metastasis and invasion in a variety of human malignancies [46]. N-cadherin, vimentin, snail, twist, and fibronectin are known as mesenchymal markers, which are closely linked to several human malignancies [47]. In addition, Snail can also inhibit the expression of other epithelial genes such as Muc1 and promote the expression of fibronectin and vimentin, which activate EMT and are associated with tumor metastasis, recurrence, and poor prognosis of breast cancer.

Niu et al. investigated the morphological and molecular changes that occur during the EMT process after bromodomain-containing protein 7 (BRD7) overexpression. BRD7 is a tumor suppressor known to inhibit cell proliferation and cell cycle progression and to induce apoptosis in breast cancer. In addition, in vitro tests indicated that BRD7 has the ability to inhibit mobility, migration and invasion of breast cancer cells [19]. At the same time, YB1 (Y-box-binding protein 1) was identified by nano-LC-MS/MS using LTQ Velos Orbitrap MS coupled to UltiMate RSLCnano LC as a new interacting BRD7 protein. It was further confirmed that EMT is a common change that occurs with altered expression of either BRD7 or YB1, and that BRD7 suppresses mesenchymal genes and activates epithelial genes [19]. Additionally, the possible contribution of annexin 1 (ANXA1) to breast tumorigenesis was investigated using stable quantitative MS proteomics based on isotope labeling. ANXA1 has been reported to promote migration and invasion of metastatic breast cancer cells as a modulator of EMT, such as phenotypic transition, through the transforming growth factor signaling pathway [18]. It has been revealed that ATRA modulates EMT of mammary tumor cells via the TGF-β and NOTCH pathways [48], and that modulation of the NOTCH1 signal transduction pathway plays a major role in ATRA activated anti-motility responses. The TGF pathway was also found to be a second signal transduction system that is essential for ATRA anti-migration. Doe at al. confirmed that the retinoic acid receptor alpha gene (RARA) regulates EMT-inducing transcription factors such as SLUG, FOXC2, ZEB1 and ZEB2, and factors activating TGF-β-SMAD signaling, including TGFBR1, TGFBR2, TGFB2 and SMAD3 [49].

Although it is widely believed that EMT contributes to metastasis, there is a lack of definitive in vivo evidence to support this theory. Some published papers report that although therapeutic inhibition of EMT might not prevent metastasis, combining chemotherapy with EMT inhibition might help to prevent the emergence of resistance [50,51]. Targeting EMT can serve as an effective strategy for cancer treatment, and EMT research will be promising in the coming years.

## 4. Natural and Synthetic Retinoid Acid Receptor Ligands and Their Role in EMT

**Figure 4:**
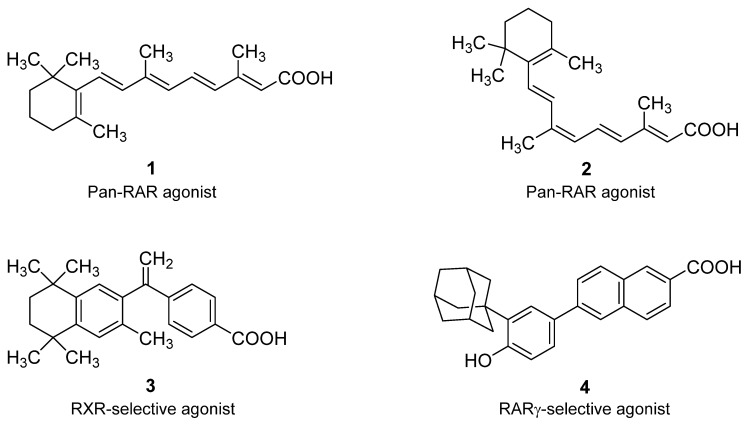
Chemical structure of selected important RAR and RXR ligands [59]. (1) All-*trans* retinoic acid (ATRA); (2) 9-*cis* retinoic acid (9cRA); (3) 4-[1-(3,5,5,8,8-pentamethyl-5,6,7,8-tetrahydronaphthalen-2-yl)ethenyl]benzoic acid (LGD1069, Bexarotene); (4) 6-[3-(1-adamantyl)-4-hydroxyphenyl]-2-naphtalene carboxylic acid (CD437).

Retinoids are natural and synthetic compounds having structural or biological activities similar to retinoic acid (RA). They include polyisoprenoid compounds containing a cyclohexenyl ring [52]. The breakthrough incomprehension of the mechanism of retinoids action brought the discovery of the superfamily of nuclear receptors comprising retinoic acid receptors (RARs) and retinoid X (RXRs) receptors [53,54]. Figure 4 presented the chemical structure of selected essential retinoids (activated by RARs) and rexinoids (engaged by RXRs). RARs and RXRs are retinoid/rexinoid inducible transcription factors that play an irreplaceable role in many tissues of higher vertebrates. They are considered to be ligand-activated, DNA-binding, trans-acting, transcription-modulating proteins [55,56,57,58]. Together with their cognate biologically active ligands, their presence in the organism is essential for many important functions, e.g., cell growth and differentiation.

Retinoids are known to inhibit carcinogenesis because they induce suspension of growth, differentiation and cause cell death in many types of cancer cells (e.g., mammary gland cancer, acute promyelocytic leukemia, neuroblastoma, gastric carcinoma, or animal and human breast tumors). They are considered to be promising anticancer drugs for a variety of types of cancer [60,61,62]. Many retinoids and rexinoids, acting through their cognate nuclear receptors, have been tested both in vitro and in vivo, using cell cultures or animal models [63,64]. In animal models, retinoids or rexinoids have been shown to suppress carcinogenesis or induce malignant cell differentiation through their cognate nuclear receptors in various tissues [65]. Because ATRA is considered the primary biologically active form of vitamin A with multiple functions in vertebrates and 9-*cis* retinoic acid (9cRA) is a high-affinity ligand for all-*trans* retinoic acid receptors, this article points out their use in chemotherapy. Altered expression of all-*trans* or 9-*cis* retinoic acid receptors may be associated with processes of malignant transformation of animal or human cells in various tissues.

In 2016, Cui et al. have investigated the effects of ATRA at different concentrations (0.1, 1.0 and 10.0 µmol/L) on the proliferation, migration, and invasion of the mouse hepatocarcinoma cell line and explored whether ATRA influences the cell phenotype and regulates EMT in the antitumor process. The authors raised a hypothesis that the effect of ATRA might be closely related to the reverse process of EMT [66]. Additionally, the study of Guan et al. added new evidence that retinoic acid isomers (ATRA and 9cRA) at different concentrations from 5.0–20.0 µmol/L inhibit pancreatic cancer cell migration and EMT through the downregulation of interleukin-6 [67].

It has also been published that ATRA can inhibit the malignant behaviors of hepatocarcinoma cells. Fang et al. investigated the effect of autophagy on the function of ATRA on hepatocarcinoma cells. Their findings show that ATRA at a concentration of 10.0 µmol/L induces autophagy and autophagic cell death through the Bcl-2/Beclin1 pathway. In addition, ATRA-induced autophagy is involved in the inhibitory effect of ATRA on the malignant behavior of hepatocarcinoma cells by reversing the EMT process [68].

### ATRA and Breast Cancer

The fact that the use of retinoids in breast cancer is in cynosure is reflected by the increasing number of scientific articles and pre-clinical as well as clinical studies. Several reviews dealing with the potential of ATRA and their derivatives in the growth and progression of breast cancer were published [64,69,70,71]. A significant part of breast carcinoma studies is based on the analysis of breast cancer cell lines. The most usually used are, namely e.g., MCF-7, T-47-D, and MDA-MB-231, comprising more than 2/3 of all abstracts of published studies. Further information on the line is given in Table 2. Cancer cell lines are advantageous because they provide an unlimited source of homogenous material, without contamination, easily cultured in standard media [72].

Because retinoic acid isomers are important therapeutic agents for many cancers, the protein composition of the highly invasive triple-negative human breast cancer cell line MDA-MB-231 after various retinoids treatments were studied and compared [80]. Three types of treatment were performed: 1.0 µmol/L ATRA, 0.1 µmol/L 9cRA, and a mixture of these two retinoids. Based on the results obtained by Flodrova and colleagues, it is believed that some of the identified proteins are associated with tumor progression, where their expression or overexpression indicates a poor prognosis. Among all identified proteins, proteins including annexin 2 (ANXA2), glyceraldehyde 3-phosphate (G3P), vimentin (VIME) and nucleophosmin (NPM) were strongly reduced by ATRA.

Some working groups have already applied the combination of 2D gel electrophoresis with mass spectrometry to distinguish the changes in the protein composition of MCF-7 human breast cancer cells induced by retinoid treatments [77,78]. The protein differences appeared when comparing the protein profile of the untreated and retinoid treated cancer cells. The significant differences between individual samples were mainly observed in cases of heat shock protein 27 and cofilin-1. Up-regulation of these proteins by ATRA could affect the process of cell migration dependent on cytoskeleton remodeling in cancer cells, which could be clinically beneficial [78].

Kamal et al. identified a group of proteins that are differentially expressed with the effect of 10^−2^ M RA in MCF-7 cells in a time-dependent manner. Using a combination of 2-D GE and MS/MS, the authors identified 35 proteins (e.g., nucleoredoxin) that can respond to RA-mediated apoptosis in breast cancer [77].

In 2020, Strouhalova et al. also analyzed and compared the protein profiles of the membrane and cytoplasmic fractions of MDA-MB-231 cells after treatment with 1.0 µmol/L ATRA [24]. Proteins such as vimentin and CD44 are linked to EMT process, were selected for this study. Decreased levels of vimentin and CD44 in the cytoplasmic, as well as membrane fraction after ATRA treatment, were confirmed. A significant result was obtained with CD44, where the protein level in the cytoplasmic fraction was almost completely suppressed. Due to the fact that CD44 is associated with resistance to treatment and poor prognosis of many cancers [81,82,83,84,85], it is highly desirable to reveal other aspects of the mechanism of action of ATRA in breast cancer. On the other hand, in many cancers, high levels of CD44 expression are not always associated with adverse outcomes [86]. Current discoveries show that different variants CD44 is expressed in human tumors, and prognosis can be estimated by the type of isoform. Other research groups analyzing the same neoplastic disease have reached conflicting conclusions about the correlation between CD44 expression and disease prognosis, probably due to inconsistent methodology [87,88].

Some studies have also examined the effect of ATRA on some solid tumors [89,90,91,92]. Although in vitro or in vitro studies indicated a chemoprotective effect of ATRA on breast, lung, and cervical cancer, clinical studies did not provide any apparent benefit. On the other hand, ATRA in combination with other drugs, has shown an advantage over ATRA-based treatment. The addition of ATRA to paclitaxel and cisplatin could increase response rates and progression-free survival in patients with advanced no small cell lung cancer. Organic arsenic melarsoprol in combination with ATRA significantly inhibited the growth of human breast and prostate cancer cells in vitro and in vivo [93].

Some biologically active derivatives, such as organotin compounds also play a role of ligands of RARs [94,95]. Some of them have been gaining growing importance in oncology [96], since they might affect a variety of nuclear receptor signaling pathways through their effect on RXR subtypes [97]. A recent study has shown that tributyltin chloride (TBT-Cl) and triphenyltin chloride (TPT-Cl) have different effects on cell proliferation and expression of apoptosis marker proteins levels in the human breast cancer MCF-7 cell line. ATRA, regardless of dose and time treatment, did not affect MCF-7 cell proliferation. On the other hand, data clearly demonstrated dose-dependent cell growth inhibition by both TBT-Cl and TPT-Cl [98].

Incubation of MDA-MB-231 cells with triorganotin compounds (either trialkyltin or triaryltin) caused decreased expression of proteins associated with either EMT or apoptosis. In addition, when MDA-MB-231 cells were treated with TBT-Cl or TPT-Cl in combination with ATRA (1.0 µmol/L), there was a further reduction in VIME, annexin 5 or nucleoside diphosphate kinase B [99]. Using iTRAQ technology, similar data have recently been reported confirming down-regulation of vimentin by newly synthesized triorganotine isothiocyanates. Triphenyltin/tributyltin isothiocyanate derivatives, both compounds, predominantly in combination with ATRA reduced the expression of VIME [100], which is a marker of mesenchymal phenotype [101,102,103]. Because EMT is characterized by down-regulation of epithelial markers and up-regulation of mesenchymal markers, the findings of this study on the combined effect of ATRA and triorganotins may be relevant information in the treatment of cancer.

## 5. Conclusions

Fundamental findings suggest that the positive therapeutic effects of ATRA observed in the clinic may also be due to its ability to reverse mesenchymal transcription programs. The reverse process, MET, allows mesenchymal cells to reverse to an epithelial phenotype and plays a key role in the metastatic spread of cancers. The ability to integrate a wide range of proteomic approaches, along with other information derived from interdisciplinary tools, opens up new interesting and promising perspectives to improve our understanding of complex processes such as EMT. In addition, understanding the entire molecular process of EMT could allow the identification of potential diagnostic markers and the selection of new therapeutic targets.

As was demonstrated above, ATRA was found to be a potential anticancer agent. At the same time, a fascinating space is emerging for the future of EMT and cancer research, where proteomics can also contribute.

## Figures and Tables

**Table 1:** An overview of some of the most common markers that demonstrate these subtypes.

Protein Name	MW (kDa)	Up/down Regulated during Cancer	Protein Function (www.uniprot.org)	References
Annexin 1 (ANX1)	38.7	UP	- potential marker of the development of breast cancer - possible role in the early events of malignant transformation	[18]
Bromodomain-containing protein 7 (BRD7)	74.1	Up/tumor suppression	- acts both as a coactivator and as a corepressor - possible role in chromatin remodeling - potential tumor suppressor in hepatocellular carcinoma	[19,20]
E-cadherin	97.5	Down	- calcium-dependent cell adhesion proteins - involved in mechanisms regulating cell-cell adhesions, mobility, and proliferation of epithelial cells	[21,22]
N-cadherin	99.8	Up	- acts as a regulator of neural stem cells quiescence by mediating anchorage of neural stem cells to ependymocytes in the adult subependymal zone - role in cell-to-cell junction formation between pancreatic beta cells and neural crest stem cells	[21,22]
β-Catenin	9.2	Up	- belongs to the cytoskeletal proteins - involved in regulation and coordination of cell-cell adhesion and gene transcription - accumulation of cytoplasmic β-catenin: useful predictor of hematogenous metastases	[23]
CD44	81.5	Up	- cell-surface receptor - role in cell-cell interactions, cell adhesion, and migration - expression is associated with resistance to therapy and poorer prognosis of many cancers - overexpression is a characteristic marker for tumorigenic cancer cells population of breast cancer, colon, pancreas, and prostate	[24,25]
Type 1 collagen	138.9	Promotes survival of human breast cancer cells by overexpressing Kv10.1 potassium and Orai1 calcium channels.	- tumor microenvironment factors - regulates proliferation, survival, migration, and invasion	[17,26]
Type IV collagen	164.0	Down	- the major structural component of glomerular basement membranes - cell adhesion function - involved in the process of tumor invasion and metastasis, including colorectal cancer and breast cancer	[27,28]
Cytokeratin 18	48.1	Down	- role in filament reorganization - may affect various cellular processes (e.g., apoptosis, cell cycle progression, and tumor cell behavior) - decreases with the progression of EMT, and is frequently used as a marker for this process	[27,29]
Class S100 of cytoskeletal proteins	9.0–13.0	Up/Down	- mainly involved in aspects of the regulation of proliferation, differentiation, apoptosis, Ca2+ homeostasis, energy metabolism - S100A2 downregulated in many cancers (associated with poor prognosis) - S100A2 upregulated in some cancers, and other functions are unclear	[17,30]
Desmin	53.5	Up	- muscle-specific type III intermediate filament essential for proper muscular structure and function - crucial role in maintaining the structure of sarcomeres, inter-connecting the Z-disks, and forming the myofibrils	[17]
Desmoplakin	331.8	Down	- downregulation in various cancers promotes tumor progression - role in carcinogenesis is still being elucidated	[27,31]
Fibroblast-specific protein 1 (S100A4)	11.7	Up/ overexpressed in a range of different tumor types	- belongs to the S100 superfamily of cytoplasmic calcium-binding proteins and can be expressed by different cell types of mesenchymal origin - role in various cellular processes, including motility, angiogenesis, cell differentiation, apoptosis, and autophagy	[17,32]
Fibronectin	2.5	Up	- fibronectins bind cell surfaces and various compounds, including collagen, fibrin, heparin, DNA, and actin - belongs to the extracellular matrix proteins - can be upregulated by SNAIL and TWIST in type 3 EMT	[33]
α5 integrin	114.5	Up	- belongs to the cell-surface proteins - receptor for fibronectin and fibrinogen - may promote tumor invasion, and higher expression of this gene may be correlated with shorter survival time in lung cancer patients	[33]
β6 integrin	85.9	Up	- belongs to the cell-surface proteins - increased β6 expression occurs in up to one-third of solid tumors, including breast cancer, lung cancer, and pancreatic cancer - not found on most normal cells - potential therapeutic target in cancer research - over-expression often correlates with poorer overall survival	[33]
Laminin 1	177.6	Down	- belongs to extracellular matrix proteins - important for adhesion, differentiation, migration, and resistance to apoptosis of various cells, including cancer cells - thought to mediate the attachment, migration, and organization of cells into tissues during embryonic development	[27]
Laminin 5	399.7	Up	- belongs to extracellular matrix proteins - highly expressed in several types of epithelial tumors - overexpression has been described in 70% of triple-negative breast carcinomas and has a role in the aggressive phenotype of some breast cancers and may provide a prognostic marker for triple-negative breast carcinoma	[33,34]
Mucin 1	122.1	Down	- can act both as an adhesion and an anti-adhesion protein - in activated T-cells, influences directly or indirectly the Ras/MAPK pathway - promotes tumor progression	[21,22]
Occludin	59.1	Down	- able to induce adhesion when expressed in cells lacking tight junctions - downregulation = common feature of EMT in tumors derived from simple epithelial cells - the decreased expression suggests that tumorigenesis is accompanied by loss of cell-cell adhesion followed by loss of differentiation and uncontrolled proliferation	[27,35]
Smooth muscle alpha-actin (α-SMA)	42.0	Up	- expressed by tumor cells carcinoma - tumor cells expressing α-SMA are predicted to be the cells having the invasive nature, tend to metastasize, and have a poorer prognosis	[17,33]
Snail	29.1	Up	- family of transcription factors - involved in the induction of the EMT, formation, and maintenance of embryonic mesoderm, growth arrest, and survival - upregulated in several cancers and associated with increased tumor migration	[33,36]
Syndecan-1	32.5	Up	- a novel molecular marker for triple-negative inflammatory breast cancer - modulates the cancer stem cell phenotype	[33,37]
Twist	21	Up	- plays an essential role in cancer metastasis - over-expression of Twist or methylation of its promoter is common in metastatic carcinomas - acts as a transcriptional regulator - inhibits myogenesis	[33,36]
Vimentin (VIME)	53.7	Up	- belongs to the cytoskeletal proteins - class-III intermediate filaments found in various non-epithelial cells, especially mesenchymal cells - highly expressed in fibroblasts, some expression in T- and B-lymphocytes, and little or no expression in Burkitt’s lymphoma cell lines - expressed in many hormone-independent mammary carcinoma cell lines	[17,36]
Y-box-binding protein 1	35.9	Reduces ovarian cancer cell proliferation	- associated with tumor and the emergence of treatment resistance - DNA- and RNA-binding protein involved in various processes	[19,38]
ZEB proteins ZEB1 ZEB2	124.1 133.8	Up Up	- transcriptional repressors - key role in solid cancer metastases by allowing cancer cells to invade and spread through transcriptional regulation of EMT - ZEB expression also associated with cancer acquisition stem cell properties and resistance to therapy - considered reliable prognostic markers of solid tumor aggressiveness	[33,36,39]
ZO-1	187.0	Down/up	- an important role in podosome formation and associated function, thus regulating cell adhesion and matrix remodeling - down-or upregulation observed in various tumors	[27,35,40]

**Table 2:** Summary of selected cell lines used for ATRA studies in human breast cancer.

Human Breast Cancer	Lines Description	References
MCF-10A	no tumorigenic	Reinhardt et al., 2018 [73]
BCM-3887	ER−, PR−, HER2−	Coyle et al., 2018 [74]
BCM-2665	ER−, PR−, HER2−	Coyle et al., 2018 [74]
BT-20	ER−, PR−, HER2−	Reinhardt et al., 2018 [73] Coyle et al., 2018 [74]
BT-474	ER+, PR+, HER2+	Reinhardt et al., 2018 [73]
DU4475	ER−, PR−, HER2−	Coyle et al., 2018 [74]
HBL-100	epithelial	Enikeev et al., 2021 [75]
HCC1187	ER−, PR−, HER2−	Coyle et al., 2018 [74]
HCC1806	ER−, PR−, HER2−	Coyle et al., 2018 [74]
HCC1937	ER−, PR−, HER2−	Coyle et al., 2018 [74] Enikeev et al., 2021 [75]
HCC1954	ER−, PR−, HER2+	Enikeev et al., 2021 [75]
HCC38	ER−, PR−, HER2−	Coyle et al., 2018 [74]
HCC70	ER−, PR−, HER2−	Coyle et al., 2018 [74]
MCF-7	ER+, PR+, HER2−	Reinhardt et al., 2018 [73] Enikeev et al., 2021 [75] Huang et al., 2019 [76] Kamal et al., 2014 [77] Flodrova et al., 2015 [78]
MDA-MB-231	ER−, PR−, HER2−	Strouhalova et al., 2020 [24] Reinhardt et al., 2018 [73] Coyle et al., 2018 [74] Enikeev et al., 2021 [75] Croker and Allan 2012 [79] Flodrova et al., 2017 [80]
MDA-MB-453	ER−, PR−, HER2−	Reinhardt et al., 2018 [73] Coyle et al., 2018 [74] Enikeev et al., 2021 [75]
MDA-MB-436	ER−, PR−, HER2−	Reinhardt et al., 2018 [73] Coyle et al., 2018 [74]
MDA-MB-468	ER−, PR−, HER2−	Coyle et al., 2018 [74] Enikeev et al., 2021 [75] Croker and Allan 2012 [79]
SK-BR-3	ER−, PR−, HER2+	Reinhardt et al., 2018 [73] Enikeev et al., 2021 [75]
SUM-149	ER−, PR−, HER2−	Coyle et al., 2018 [74]
SUM-159	ER−, PR−, HER2−	Coyle et al., 2018 [74]
T47D	ER+, PR+, HER2−	Reinhardt et al., 2018 [73] Enikeev et al., 2021 [75] Huang et al., 2019 [76]
ZR-75-1	ER+, PR−, HER2−	Reinhardt et al., 2018 [73]

## Data Availability

Not applicable.

## Abbreviations

ANXA1	Annexin 1
ANXA2	Annexin 2
APL	Acute promyelocytic leukemia
ATRA	All-*trans* retinoic acid
BRD7	Bromodomain-containing protein 7
CDK1	Cyclin-dependent kinase 1
9cRA	9-*cis* retinoic acid
EMT	Epithelial-mesenchymal transition
ER	Estrogen receptor
ERBB2	Receptor tyrosine kinase 2
FGF	Fibroblast growth factors
FOXC2	Forkhead box protein C2
FSP1	Fibroblast specific protein 1
G3P	Glyceraldehyde 3-phosphate
Her2	Human epidermal growth factor receptor 2
HGF	Hepatocyte growth factor
INPP4B	Inositol polyphosphate-4-phosphatase, type II
MET	Mesenchymal-epithelial transition
MS	Mass spectrometry
NB	Neuroblastoma
NPM	Nucleophosmin
PR	Progesterone receptor
RA	Retinoic acid
RARs	Retinoic acid receptors
RXRs	Retinoid X receptors
α-SMA	Smooth muscle alpha-actin
TBT-Cl	Tributyltin chloride
TGF-β	Transforming growth factor beta
TPT-Cl	Triphenyltin chloride
VIME	Vimentin
YB1	Y-box-binding protein 1
ZEB	Zinc finger E-box-binding homeobox
